# The Impact of Practice Environment and Resilience on Burnout among Clinical Nurses in a Tertiary Hospital Setting

**DOI:** 10.3390/ijerph18052500

**Published:** 2021-03-03

**Authors:** Dzifa Dordunoo, Minjeong An, Min Sun Chu, Eun Ja Yeun, Yoon Young Hwang, Miran Kim, Yeonhu Lee

**Affiliations:** 1 School of Nursing, University of Victoria, Victoria, BC V8P 5C2, Canada; dzifa@uvic.ca; 2 Interdisciplinary Program of Arts & Design Technology, College of Nursing, Chonnam National University, 160 Baekseoro, Donggu, Gwangju 61469, Korea; sagijuju@naver.com; 3 Department of Nursing, Seoul Women’s College of Nursing, 38 Ganhodaero, Seodaemungu, Seoul 03617, Korea; hwangyoonyoung@naver.com; 4 Department of Nursing, Konkuk University, 268 Chungwon-Daero, Chungjusi 27478, Korea; eunice@kku.ac.kr; 5 Department of Nursing, Songwon University, 73 Songamro, Namgu, Gwangju 61756, Korea; ing7188@gmail.com

**Keywords:** burnout, nurses, practice environment, resilience

## Abstract

The purpose of this study was to examine practice environment, resilience, and burnout and to identify the impacts of practice environment and resilience on burnout among clinical nurses working at a tertiary hospital. A cross-sectional secondary data analysis was conducted using a convenience sample of 199 nurses. The nurses completed survey questionnaires regarding practice environment, resilience, and burnout. The majority of the nurses were below the age of 30, single, and worked in medical-surgical wards. Approximately, 92% of the nurses reported moderate to high burnout, with a mean practice environment score of 2.54 ± 0.34 and resilience score of 22.01 ± 5.69. Practice environment and resilience were higher in the low level of burnout than in the moderate to high level of burnout. After controlling for demographic and occupational characteristics, resilience and nursing foundations for quality of care were significant predictors of burnout (OR = 0.71, *p* = 0.001; OR = 0.01, *p* = 0.036, respectively), explaining 65.7% of the variance. In a mixed practice environment, increased resilience and nursing foundations for quality of care lowered nurses’ burnout. Our findings suggest that interventions focused on enhancing individual resilience and practice environment and building better nursing foundations for quality of care should be developed and provided to alleviate burnout in clinical nurses working at tertiary hospitals. Nursing and hospital administrators should consider the importance of practice environment and resilience in nurses in developing interventions to decrease burnout.

## 1. Introduction

In 2017, the number of clinical nursing personnel, such as nurses and nursing assistants, in Korea was approximately 6.9 per 1000 people, which was 2.1 lower than the average of 9.0 from other countries of the Organization for Economic Cooperation and Development (OECD). This number (6.9) was an increase from the 4.8 per 1000 Korean clinical nursing workforce in 2012; however, it is still low and below the OECD average [1]. The lack of nurses also hinders nursing work, leading to negative consequences, such as lowering the quality of care through an increase in patients complaints, patients’ falls, infection rate, and medication errors [2]. In addition, this shortage interferes with the physical and mental health of individual nurses [3,4], causes burnout, and acts as a major cause of job turnover [5,6]. Burnout is a response to work-related personal stressful experiences, but, at the same time, it is influenced by emotional strains or stresses formed in the situational relationship between work providers and recipients, and work providers and co-workers or family members [7]. In order to manage nurses’ burnout appropriately, both individual and organizational factors that influence burnout should be considered [8].

The practice environment not only refers to a simple physical environment but also a concept that includes peer interaction and institutional policy [9], and it is closely related to burnout in nurses. According to the existing study findings, since the practice environment acts as an external environment for nurses, if the working environment is good, job dissatisfaction and burnout will decrease, the intention to stay in the hospital will increase, and the quality of patient care will improve [5,10]. Contrarily, inadequate or negative work environments characterized by low leadership competencies, lack of nursing manpower, and excessive stress factors act as obstacles to qualitative nursing performance, increasing their intention to turnover, alongside depression, and burnout [10]. An inadequate work environment causes health/safety problems and reduced work performance in nurses and poor quality of care of patients; thus, efforts to maintain the practice environment properly are necessary, and research is required to examine the conditions of practice environments [10]. Some previous studies in Korea investigated the practice environment as a variable, but only the average score was confirmed [8,10]. Using sub-scales could be an alternative way to understand and communicate to nurses while evaluating the practice environment; however, studies that specifically investigated the practice environment by sub-scales are insufficient. Therefore, it is necessary to examine the practice environment in tertiary hospitals, which care for and treat the majority of inpatients and patients with high disease severity, in detail. It is also important to identify important factors for its improvement.

Resilience, which is a personal attribute of a nurse, is an inherent potential ability that enables them to effectively cope with work-related stress or workplace adversity situations [5,11]. It is a complex and dynamic concept relating to the power of recovery over time and, though it is natural, it can be nurtured through experience and education [12,13]. Resilience activates the positive internal energy of nurses [11,14,15], and it is a major attribute that affects the quality of care and organizational efficiency [16]. Nurses with higher resilience levels actively utilize internal and external resources to overcome difficulties and minimize the negative effects of stress and to improve sleep quality and well-being, resulting in good performance in nursing work [17,18]. However, when the level of resilience is low in nurses, the ability to manage stress effectively decreases, leading to negative consequences such as burnout and increased turnover intent [18,19]. Many studies on resilience in nurses have been conducted among those working in general hospitals [8,10,20], long-term care hospitals (LTCHs) [21,22], or in mixed settings, that is general and tertiary hospitals [16]. However, studies about nurses treating patients with high disease severity at tertiary hospitals are insufficient; thus, studies in this area should be conducted to provide fundamental evidence to help prevent nurse burnout and to develop effective burnout prevention measures for nurses.

### 1.1. Aim

This study aimed to examine practice environment (as an organizational factor), resilience (as an individual factor), and burnout and aimed to identify the impacts of these factors on burnout among clinical nurses working at a tertiary hospital.

### 1.2. Hypotheses

Summarizing the literature review findings, we propose the following two hypotheses.

**Hypothesis** **1.** *Favorable practice environment is negatively associated with burnout among nurses working at a tertiary hospital.*

**Hypothesis** **2.** *Higher resilience is negatively associated with burnout among nurses working at a tertiary hospital.*

## 2. Materials and Methods

### 2.1. Study Design, Sample, and Procedure

This study used the cross-sectional secondary data analysis of a previously published descriptive correlational study on the factors affecting the quality of nursing service among clinical nurses in Korea [23]. In the original study, participants were recruited from a tertiary hospital with 1033 beds located in the city of Gwangju, Korea. Flyers were sent out using an email link through the hospital’s nursing intranet three times per month for approximately three months. A total of 991 nurses were invited to participate in this study, and approximately 20% (*n* = 199) responded. After the respondents signed up by email or phone, consent forms and surveys were distributed by mail in enclosed, resealable envelopes. Research assistants visited the wards of each participating nurse on a scheduled day to collect the sealed envelopes that had been placed in designated places. We classified a survey questionnaire with more than 80% of the questions answered as completed. Participation was completely voluntary, and participants could withdraw at any time, without discrimination. Participants received stationery goods worth $5 and a thank-you note for their participation. Ethics clearance for the current study was obtained from the University of Victoria, Canada (18-040).

### 2.2. Measurements

#### 2.2.1. Practice Environment

Practice environment was assessed using the Korean version of the Nursing Work Index [24,25]. The Korean version of the scale has 29 items and consists of five sub-scales including nurse participation in hospital affairs, nursing foundations for quality of care, nurse manager ability, leadership, and support of nurses, staffing and resource adequacy, and collegial nurse–physician relations. The responses were aligned to a four-point Likert scale (1, strongly disagree; 4, strongly agree), with higher scores indicating more favorable practice environments. Values above 2.5 indicated general agreement and the scale was classified into three categories according to the number of sub-scales: favorable (all or four out of five sub-scales), mixed (two to three out of five sub-scales), and unfavorable (one or none of the five sub-scales) [26]. The internal consistency of the overall scale was 0.93 for the original scale and 0.92 in this study, and it ranged from 0.80 to 0.84 for the original scale [22] and from 0.79 to 0.86 for the sub-scales.

#### 2.2.2. Resilience

Resilience was measured using the Korean version of the 10-item Connor Davidson Resilience Scale [26]. The scale consists of five sub-scales including hardiness, tolerance of negative affect, optimism, social support, and spirituality. Participants were asked to rate how true each statement (i.e., able to adapt to change) had been for the past month on a five-point Likert scale (0, not at all true; 4, true nearly all the time). Higher scores (maximum of 40) indicated greater resilience. The internal consistency of the scale was 0.93 for the original scale [23] and 0.94 in this study.

#### 2.2.3. Burnout

Burnout was measured using 10 items from the Professional Quality of Life Scale (PQOL). The PQOL scale consists of two broad categories: compassion satisfaction and compassion fatigue. Compassion fatigue is further divided into burnout and secondary traumatic stress [27]. Burnout is one of the elements included in the negative impact of care known as compassion fatigue. It is associated with feelings of hopelessness and difficulties in dealing with work. Questions were asked about experiences of burnout over the previous 30 days on a five-point Likert scale (1 = never, 5 = very often), and the total scores were calculated as a sum of the 10 items, with higher scores indicating a higher level of burnout. The burnout total score was classified into three categories: low (22 or less), moderate (between 23 and 41), and high (42 or more) [24]. The internal consistency of the scale was 0.75 for the original scale [24] and 0.77 in this study.

The following demographic characteristics were among those included in the assessment: age (in years), marital status (single vs. married), and education level (associate degree or bachelor vs. master’s degree and higher). Occupational characteristics included working position (staff nurse vs. head nurse), career length (in years), ward type (medical vs. surgical), and perceived health status (bad vs. good).

### 2.3. Data Analysis

Data were analyzed using the Statistical Package for Social Science (SPSS) version 26 (IBM, Armonk, New York, NY, USA). Descriptive analysis using means, standard deviations, frequencies, and percentages were computed to explain sample and variables. Independent t-tests, analysis of variance (ANOVA), or chi-squared tests were performed to compare practice environment, resilience, and burnout, according to demographic and occupational characteristics. Finally, a hierarchical logistic regression analysis was performed to explore the impact of practice environment and resilience on burnout, after controlling for demographic and occupational factors. Demographic and occupational characteristics showing differences in burnout were entered into Model 1, followed by practice environment factors (sub-scales of practice environment, Model 2), and resilience levels (Model 3).

## 3. Results

### 3.1. Demographic and Occupational Characteristics

Of the 199 participants, approximately half of the nurses were below the age of 30 (mean age, 30.40 years). Majority of the participants were single (*n* = 134, 67.3%) and had completed a diploma or bachelor’s degree (*n* = 181, 91.0%). Most of them were staff nurses (*n* = 186, 93.5%) and have worked for at least three years or more as a nurse (*n* = 140, 70.4%). Currently, more than half of the nurses worked at the medical ward (*n* = 108, 54.3%) and reported bad health status (*n* = 108, 55.7%) (Table 1).

### 3.2. Practice Environment, Resilience, and Burnout

Overall, the mean score regarding the practice environment was 2.54 ± 0.34, and it was classified as a mixed environment, with three sub-scales and a mean score greater than 2.5. The two sub-scales with the highest scores were nurse manager ability, leadership, and support of nurses and nursing foundations for quality of care (2.90 ± 0.50, 2.80 ± 0.33, respectively), while the staffing and resource adequacy subscale had the lowest score (1.99 ± 0.54). The mean resilience score was 22.01 ± 5.69, and most of the participants indicated moderate to high burnout rates (*n* = 183, 92.0%) (Table 1).

### 3.3. Comparisons of Practice Environment, Resilience, and Burnout, by Demographic and Occupational Characteristics

Practice environment showed significant differences by age group, working position, and perceived health status (Table 2). Specifically, nurses aged 40 years and older, head nurses, and/or nurses with good health status found their practice environment to be more favorable than nurses younger than 40, staff nurses, and/or those with bad health status did (*F* = 3.27, *p* = 0.040; *t* = −3.66, *p* < 0.001; *t* = −2.52, *p* = 0.013, respectively). Resilience showed significant differences by age group, marital status, education level, working position, career length, and perceived health status. Resilience was significantly higher in nurses aged 40 years and older, those with master’s degree or higher, those with good health status (F = 10.30, *p* < 0.001; t = −4.38, *p* < 0.001; t = −2.00, *p* = 0.047, respectively), those who were married, and head nurses (t = −4.14, *p* < 0.001; t = −5.02, *p* < 0.001, respectively) than it was in their respective counterparts. Characteristically, higher resilience levels were reported in participants who had worked for at least three years and longer than in those who had worked for less than three years (t = −3.04, *p* = 0.003). Practice environment and resilience showed significant differences by burnout level. In other words, each subscale of practice environment and resilience was higher in the low level of burnout than in the moderate to high levels of burnout (nurse participation in hospital affairs: t = 3.13, *p* = 0.002; nursing foundations for quality of care: t = 5.47, *p* < 0.001; nurse manager ability, leadership, and support of nurses: t = 2.29, *p* = 0.023; staffing and resource adequacy: t = 2.16, *p* = 0.032; collegial nurse–physician relations: t = 4.40, *p* < 0.001; resilience: t = 7.92, *p* < 0.001, respectively).

### 3.4. Impact of Practice Environment and Resilience on Burnout in Clinical Nurses

A series of binary logistic regression analyses was conducted to examine the impact of practice environment and resilience on nurses’ level of burnout (mild vs. moderate to high) after controlling for demographic and occupational characteristics (Table 3). In Model 1, demographic and occupational characteristics that showed significant differences in burnout level were entered as covariates, and education level and job position were significant predictors, explaining 34.9% of the variance in burnout level (Nagelkerke R^2^ = 0.349). In Model 2, practice environment was added to Model 1, and nursing foundations for quality of care was identified as a significant factor affecting burnout level, after controlling for the demographic and occupational factors, explaining 51.5% of the variance (Nagelkerke R^2^ = 0.515). In Model 3, resilience was added to Model 2, and nursing foundations for quality of care, which is a sub-scale of the practice environment, and resilience predicted less likelihood of moderate to high level of burnout, after controlling for the covariates, explaining 65.7% of the variance in burnout level (Nagelkerke R^2^ = 0.657). The findings showed that 99% and 29% of the nurses were less likely to have moderate to high level of burnout, with every one-point increase in the score of nursing foundations for quality of care (OR = 0.01, *p* = 0.036) and in the resilience score (OR = 0.71, *p* = 0.001), respectively. These results supported hypotheses 1 and 2.

## 4. Discussion

In this study, we sought to explore how practice environment and resilience contribute to burnout after controlling for socio-demographic and occupational characteristics. Our results revealed that resilience and nursing foundations for quality of care, which is a sub-scale of the practice environment, are significant predictors of burnout. The findings suggest that improving nursing foundations for quality of care at the organizational level and resilience at the individual level could decrease burnout in clinical nurses working at the tertiary hospital.

In our study, the practice environment was mixed, and only nursing foundations for quality of care in the practice environment was a significant predictor of burnout. Among the five sub-scales of the practice environment, the score of nursing foundations for quality of care as well as the scores of nurse manager ability, leadership, and support of nurses were high. It is thought that qualitative nursing care systems are well equipped and the quality and level of nurse managers are guaranteed. However, the average scores of the two sub-scales were lower than those of the Magnet Hospital scores, which was awarded as Magnet designation hospital by the American Nurses Credentialing Center [28], implying that more efforts to improve the practice environment should be made by hospitals and nursing administrators for nurses to find their workplace favorable. Among the sub-scales, participation of nurses in hospital affairs and staffing and resource adequacy had average scores of less than 2.5. In previous studies, the average scores of nurse participation in hospital affairs, and staffing and resource adequacy, were reported to be the lowest [29,30]. This is probably because the level of nurses’ participation in hospital administration was limited and nurses believe that institutional support was insufficient in the practice environment [22]. Especially, inadequate staffing is a global problem and a major concern in the practice environment of nurses [30]. Since a higher nurse-to-patient ratio increases nurse burnout and dissatisfaction, leading to poor quality of care, the establishment of better practice environments consequently is beneficial to both nurses and patients [5]. The fact that Korea’s patient-to-nurse ratio is higher than that of other countries [1] is considered to have had an effect [29,30].

Nursing foundations for quality of care, which is a sub-scale of the practice environment, and resilience, were statistically significant predictors of burnout. Because nursing foundations for quality of care is associated with increased job satisfaction, intention to stay at the hospital, and quality of care [31], as nurses’ internal and work satisfaction increase, burnout rates reduce. Therefore, it is imperative to find a method to identify and promote the facilitating factors of nursing foundations for quality of care, and an administrative system needs to be established for continuous improvement. A favorable practice environment is an essential factor that lowers the burnout rate in nurses and increases job satisfaction and the intention of retention, thus enabling high-quality nursing [31]. In addition, hospitals with mixed work environments had lower burnout rates, job dissatisfaction, and intention to leave than hospitals with poor work environments among nurses, and these factors were higher when compared with hospitals with favorable work environments [30]. Therefore, to improve mixed work environments, policymakers, hospital managers, and nursing department managers need to reinforce the institution’s support policy for the working environment while considering hospital characteristics, patient characteristics, and the current status of nursing department personnel.

The findings of a previous study reported that resilience reduced burnout occurrence [32]. Resilience is an important process for lowering the impact of workplace stress and it prevents poor psychological outcomes [33]. It is a concept that combines individual nature and situational factors; individual nature is difficult to change, but situational factors can be modified [12,16]. In addition, resilience can be developed through appropriate training programs and supportive social networks that help reduce one’s vulnerability [11,16,33]. Therefore, it is necessary to provide support to strengthen internal resources by building resilience to reduce burnout rates in nurses. Furthermore, it is necessary to provide psychological support, education, and psychosocial health programs at the nursing department level and/or workplace organization level as an external resource to enhance nurse resilience [11,15,32]. In other words, resilience could be fostered through focused interventions including formal and consistent resilience education programs, social support, and meaningful recognition [12,13,34]. Thus, nursing administrators should develop and provide appropriate resilience-enhancement programs to help nurses identify their job-related stressors, know their personal triggers, and improve their coping skills. These efforts could result in decreases in burnout and improvements in patients’ outcomes.

### 4.1. Implications

The findings indicate that a favorable practice environment and higher resilience were significantly associated with a lower level of burnout. Building a favorable practice environment and resilience is essential for the quality of nursing, especially at tertiary hospitals. Nursing administrators should therefore consider the importance of practice environment and resilience in developing interventions to decrease burnout in hospitals. Policymakers should stimulate nursing and/or hospital administrators to plan and provide strategies to improve practice environment and resilience among nurses.

### 4.2. Limitations

There are some limitations to this study. First, this study was conducted using a convenient sampling method to collect data from only one tertiary hospital; thus, it is difficult to generalize the study findings. Therefore, further studies should be conducted, increasing the number and types of hospitals and including other regions in Korea. Second, this was a cross-sectional study; thus, it is difficult to explain a causal relationship of the findings. Therefore, further studies should be conducted to examine the causal relationship using a longitudinal study design.

## 5. Conclusions

In examining the factors influencing burnout in nurses working in a tertiary hospital, nursing foundations for quality of care and resilience were identified as significant influencing factors on burnout level. Our findings suggest that interventions focused on enhancing individual resilience and overall practice environment, and building better nursing foundations for quality of care, should be developed and provided to alleviate burnout in clinical nurses working at tertiary hospitals.

## Figures and Tables

**Table 1:** Demographic and occupational characteristics and outcome variables of the participants (*n* = 199).

Characteristics	Categories	Mean ± SD or *n* (%)	Range
Age (year) *		30.40 ± 6.58	23–53
	23–29	107 (54.9)	
	30–39	69 (35.4)	
	≥40	19 (9.7)	
Marital status	Single	134 (67.3)	
	Married	65 (32.7)	
Education level	≤Bachelor	181(91.0)	
	≥Master’s degree	18 (9.0)	
Position	Staff nurse	186 (93.5)	
	Head nurse	13 (6.5)	
Career length (year)	<3 years	59 (29.6)	0.42–31.67
	≥3 years	140 (70.4)	
Ward type	Medical	108 (54.3)	
	Surgical	91 (45.7)	
Perceived	Bad	108 (55.7)	
health status **	Good	86 (44.3)	
Practice	Total	2.54 ± 0.34	1.66–3.62
environment	Nurse participation in hospital affairs	2.41 ± 0.46	1.11–3.56
	Nursing foundations for quality of care	2.80 ± 0.33	1.89–3.89
	Nurse manager ability, leadership, and support of nurses	2.90 ± 0.50	1.25–4.00
	Staffing and resource adequacy	1.99 ± 0.54	1.00–3.50
	Collegial nurse–physician relations	2.57 ± 0.57	1.00–4.00
Resilience		22.01 ± 5.69	8.00–40.00
Burnout	Total	28.50 ± 4.79	11.00–43.00
	Low (≤22)	16 (8.0)	
	Moderate (23–41)	182 (91.5)	
	High (≥42)	1 (0.5)	

Note: * missing = 4; ** missing = 5.

**Table 2:** Differences in outcome variables by demographic and occupational characteristics (*n* = 199).

Characteristics	Categories	Practice Environment			Resilience			Burnout			
		Mean ± SD	t/F	*p*	Mean ± SD	t/F	*p*	Low (*n*, %)	Moderate-High (*n*, %)	X^2^	*p*
Age	<30 ^a^	2.53 ± 0.32	3.27	0.040	20.73 ± 4.65	10.30	<0.001	6 (37.5)	101 (56.4)	15.27	<0.001
(year)	30–39 ^b^	2.52 ± 0.32		(a,b < c)	22.49 ± 6.00		(a,b < c)	4 (25.0)	65 (36.3)		
	≥40 ^c^	2.73 ± 0.41			26.74 ± 7.42			6 (37.5)	13 (7.3)		
Marital	Single	2.51 ± 0.32	−1.97	0.050	20.78 ± 4.81	−4.14	<0.001	6 (37.5)	128 (69.9)	7.04	0.008
status	Married	2.61 ±0.37			24.54 ± 6.52			10(62.5)	55 (30.1)		
Education level	≤Bachelor	2.52 ± 0.32	−1.84	0.081	21.33 ± 5.08	−4.38	<0.001	8 (50.0)	173 (94.5)	35.47 *	<0.001
	≥Master’s	2.72 ± 0.45			28.83 ± 7.09			8 (50.0)	10 (5.5)		
Position	Staff nurse	2.52 ± 0.32	−3.66	<0.001	21.50 ± 5.21	−5.02	<0.001	9 (56.3)	177 (96.7)	39.47 *	<0.001
	Head nurse	2.86 ± 0.43			29.23 ± 7.51			7 (43.8)	6 (3.3)		
Career length	<3 years	2.58 ± 0.28	1.14	0.257	20.39 ± 4.26	−3.04	0.003	3 (18.8)	56 (30.6)	0.99 *	0.320
(year)	≥3 years	2.52 ± 0.36			22.69 ± 6.08			13 (81.3)	127 (69.4)		
Ward type	Medical	2.56 ± 0.36	0.66	0.511	21.37 ± 6.08	−1.72	0.087	7 (43.8)	101 (55.2)	0.78	0.378
	Surgical	2.52 ± 0.31			22.76 ± 5.13			9 (56.3)	82 (44.8)		
Health	Bad	2.49 ± 0.34	−2.52	0.013	21.31 ± 5.60	−2.00	0.047	7 (43.8)	101 (56.7)	1.00	0.316
status	Good	2.61 ± 0.32			22.94 ± 5.76			9 (56.3)	77 (43.3)		
								**Low** **Mean ± SD**	**Moderate-High** **Mean ± SD**	**T**	***p***
Practice environment	NPHA							2.75 ± 0.50	2.38 ± 0.45	3.13	0.002
	NFQC							3.20 ± 0.31	2.76 ± 0.31	5.47	<0.001
	NMA							3.17 ± 0.42	2.87 ± 0.50	2.29	0.023
	SRA							2.27 ± 0.61	1.97 ± 0.53	2.16	0.032
	CNPR							3.15 ± 0.61	2.52 ± 0.54	4.40	<0.001
Resilience								31.44 ± 5.38	21.18 ± 4.94	7.92	<0.001

a, b, and c: indicate pairwise comparisons performing ANOVA. *: indicate Fisher’s exact test results. Abbreviations: CNPR, collegial nurse–physician relations; NFQC, nursing foundations for quality of care; NMA, nurse manager ability, leadership and support of nurses; NPHA, nurse participation in hospital affairs; PES, practice environment scale total score; SRA, staffing and resource adequacy.

**Table 3:** Predictors of burnout among clinical nurses working at a tertiary hospital.

	Predictors	Model 1				Model 2				Model 3			
		b	OR	95% CI	*p*	b	OR	95% CI	*p*	b	OR	95% CI	*p*
Demographic and occupational factors	Constant	1.15	3.14		0.058	14.59	217,381.03		0.003	21.69	2,629,399,855		0.002
	Age, <40	0.66	1.93	0.20, 18.37	0.569	0.51	1.66	0.17, 16.41	0.665	0.20	1.22	0.10, 14.56	0.874
	Age, ≥40	0.69	1.99	0.16, 24.87	0.593	0.91	2.48	0.05, 128.04	0.652	1.69	5.41	0.02, 1570.56	0.560
	Marital, married	−0.18	0.84	0.09, 7.72	.876	1.23	3.42	0.14, 81.23	0.447	2.29	9.86	0.17, 584.66	0.272
	Education, ≥master’s	−2.54	0.08	0.01, 0.45	0.004	−3.39	0.03	<0.01, 0.48	0.012	−2.52	0.08	<0.01, 1.93	0.120
	Position, head nurse	−2.60	0.07	0.01, 0.62	0.016	−2.64	0.07	<0.01, 2.94	0.164	−2.81	0.06	<0.01, 10.06	0.282
Practice environment factor	NPHA					1.81	6.11	0.68, 55.14	0.107	1.92	6.80	0.51, 90.85	0.147
	NFQC					−5.05	0.01	<0.01, 0.21	0.004	−4.36	0.01	<0.01, 0.75	0.036
	NMA					0.36	1.44	0.24, 8.68	0.692	−0.10	0.90	0.11, 7.65	0.924
	SRA					−0.55	0.58	0.10, 3.23	0.533	−0.06	0.94	0.16, 5.76	0.950
	CNPR					−1.10	0.33	0.08, 1.43	0.139	−0.94	0.39	0.06, 2.44	0.315
Individual factor	Resilience									−0.34	0.71	0.58, 0.87	0.001
x^2^ (*p*)		2.23 (0.525)				26.33 (0.001)				15.93 (0.043)			
Nagelkerke R^2^		0.349				0.515				0.657			

Reference: age, <30; marital status, single; education, ≤bachelor; position, staff nurse; abbreviations: CI, confidence interval; CNPR, collegial nurse–physician relations; NFQC, nursing foundations for quality of care; NMA, nurse manager ability, leadership and support of nurses; NPHA, nurse participation in hospital affairs; SRA, staffing and resource adequacy.

## Data Availability

The data presented in this study are available on request from the corresponding author. The data are not publicly available due to ethical issue.
